# SARS-CoV-2 Genome Sequence Obtained from Ethiopia

**DOI:** 10.1128/mra.01182-21

**Published:** 2022-02-03

**Authors:** Molalegne Bitew, Getnet Hailu, Yakob Gebregziabher Tsegay, Keyru Tuki, Kominist Asmamaw, Kassahun Tesfaye, Hailu Dadi, Emanuele Orsini, Simeone Dal Monego, Danilo Licastro, Alessandro Marcello

**Affiliations:** a Ethiopia Biotechnology Institute (EBTI), Addis Ababa, Ethiopia; b Addis Ababa University, Aklilu Lemma Institute of Pathobiology (ALPB), Addis Ababa, Ethiopia; c Department of Medical Biotechnology, Institute of Biotechnology, University of Gondar, Gondar, Ethiopia; d AREA Science Park Padriciano, Trieste, Italy; e Laboratory of Molecular Virology, International Centre for Genetic Engineering and Biotechnology (ICGEB), Trieste, Italy; DOE Joint Genome Institute

## Abstract

The coding-complete severe acute respiratory syndrome coronavirus 2 (SARS-CoV-2) genome sequences from 15 nasopharyngeal swabs collected in Addis Ababa, Ethiopia, during the period from December 2020 to March 2021 were determined using Illumina MiSeq technology. A sequence analysis identified that the B.1 SARS-CoV-2 lineage was most prevalent with the worrying emergence of B.1.1.7 in June 2021.

## ANNOUNCEMENT

The severe acute respiratory syndrome coronavirus 2 (SARS-CoV-2) outbreak began in Wuhan, Hubei Province, China, in late December 2019 and spread worldwide as the worst viral pandemic in recent times (1). SARS-CoV-2 is the aetiologic agent of COVID-19, which could lead to severe pneumonia (2). SARS-CoV-2 is an enveloped virus belonging to the *Betacoronavirus* genus of the *Coronaviridae* family of RNA viruses with a single-stranded genome of positive polarity. Since the first sequence of the SARS-CoV-2 complete genome was reported in early December 2019, an exponentially increasing number of virus sequences has been reported, with over 2,612,459 complete genome sequences stored in the Global initiative on sharing all influenza data (GISAID) database to date (accessed on 8 April 2021) (3).

Ethiopia in sub-Saharan East Africa has had 306,810 confirmed COVID-19 cases and 4,675 deaths out of the total of 3,239,416 individuals tested by 4 September 2021. There is a strong demand to know whether variants of concern are circulating in this country to inform authorities and to guide public health interventions (4). However, only five sequences from Ethiopia were available on public databases from December 2020 to March 2021, reinforcing the urgent need to increase molecular surveillance (5).

A total of 15 nasopharyngeal swab samples were collected from COVID-19 patients and asymptomatic individuals in Addis Ababa, Ethiopia. Samples were collected from the period of December 2020 to March 2021 at different hospitals, and reverse transcription-quantitative PCR (qRT-PCR) detection was carried out at the Ethiopia Biotechnology Institute (EBTI). Nucleic acids were extracted using an RNA/DNA purification kit (Daan Gene Co., Ltd. of Sun Yat-sen University, Guangzhou, China) according to the manufacturer’s protocol and then treated with DNase. RNA was then suspended in RNase-free water, and then the RNA extracts were purified using an RNA clean and concentrator kit (Zymo Research, CA, USA) (6). The diagnostic detection of SARS-CoV-2 RNA was performed with the Beijing Genomic Institute (BGI) real-time fluorescent reverse transcriptase PCR (RT-qPCR) detection kit of SARS-CoV-2 (BGI, China) with primers targeting the ORF 1ab gene (5) A total of 15 samples with cycle threshold (*C_T_*) values of <30 were selected randomly for high-throughput genome sequencing. This technique is based mainly on PCR amplification of the genome (7). A Qubit 2.0 fluorimeter (Thermo Fisher Scientific, MA, USA) and Agilent 2100 bioanalyzer (Agilent Technologies, CA, USA) were used to assess the RNA quantities and qualities, respectively. From the total RNA, 100 ng was processed by the Swift Amplicon SARS-CoV-2 research panel for genome amplification and library preparation (Swift Bioscience, USA). High-throughput sequencing was conducted using an Illumina MiSeq sequencer following the standard protocol for paired-end 150-bp reads. The sequencing and data analysis were done at the ARGO Open Lab Platform for Genome Sequencing of the AREA Science Park and at the International Center for Genetic Engineering and Biotechnology (ICGEB), Trieste, Italy. All tools were run with default parameters unless otherwise specified. The raw sequence data were quality controlled using FastQC v0.11.9 (https://www.bioinformatics.babraham.ac.uk/projects/fastqc/). Genome assembly was conducted using dedicated Swift dockerized data analysis guidelines (7, 8).

The study was approved by Addis Ababa University, Aklilu Lemma Institute of Pathobiology (ALIP) institutional review board with protocol number ALIPB IRB/52/2013/21. Data were collected after permission was obtained from the institute. All the information obtained from the study participants was kept confidential.

For each sample, sequencing parameters (GC content [%], complete length, genome coverage, and number of raw reads) are listed in Table 1. Briefly, genome length across the samples was 29,817 to 29,878 bp; genome coverage was 18,000× to 22,000× for samples S1 to S10 and 3,000× to 5,000× for samples S11 to S15.

**TABLE 1:** Sequencing parameters and phylogenetic traits of sequenced genomes

Sample ID	GISAID ID	Accession no.	SRA accession no.	GC content (%)	Complete length (bp)	SARS-CoV-2 coverage (×)	No. of raw reads (millions)	Amino acid substitutions	Nexstrain clade	PANGO^*a*^ lineage
hCoV-19/Ethiopia/ICGEB-S1/2021	EPI_ISL_1897644	OL840802	SRX13137980	42.6	29,878	21,793	3.6	Spike D614G, Spike E180Q, Spike M1229I, Spike N501Y, N D377Y, N S194L, NS3 Q57H, NSP2 A31V, NSP2 L24F, NSP3 G1001S, NSP3 T350I, NSP3 V521I, NSP5 S284G, NSP12 L648F, NSP12 P323L, NSP13 T588A, NSP13 Y277C, NSP15 K70R	20A	B.1.480
hCoV-19/Ethiopia/ICGEB-S2/2021	EPI_ISL_1897646	OL840803	SRX13137981	43.3	29,878	21,521	3.8	Spike D614G, Spike E180Q, Spike E309Q, Spike M1229I, Spike N501Y, N D377Y, N S194L, NS3 Q57H, NSP2 L24F, NSP3 K1182R, NSP3 S769L, NSP3 V521I, NSP5 S284G, NSP6 A186T, NSP12 L648F, NSP12 P323L, NSP13 P82S, NSP13 Y277C	20A	B.1.480
hCoV-19/Ethiopia/ICGEB-S3/2021	EPI_ISL_1897648	OL840804	SRX13137982	43.3	29,878	21,621	3.8	Spike D614G, N P199L, NS3 Q57H, NSP2 E264K, NSP2 F499L, NSP12 P323L, NSP12 T26I, NSP13 E341D	20A	B.1
hCoV-19/Ethiopia/ICGEB-S4/2021	EPI_ISL_1897884	OL840805	SRX13137983	43.5	29,877	18,606	3.2	Spike A570D, Spike D614G, Spike D1118H, Spike N501Y, Spike P681H, Spike S982A, Spike T716I, N D3L, N G204R, N R203K, N S235F, NS3 V163L, NS8 Q27stop, NS8 R52I, NS8 Y73C, NSP1 E2K, NSP3 A890D, NSP3 G307C, NSP3 I1412T, NSP3 P141S, NSP3 T183I, NSP10 T12I, NSP12 P323L	20I/501Y.V1	B.1.1.7
hCoV-19/Ethiopia/ICGEB-S5/2021	EPI_ISL_1898258	OL840806	SRX13137984	42.8	29,871	21,735	3.8	Spike D614G, Spike N440K, Spike S13T, N P199L, NS3 Q57H, NS3 S171L, NSP2 F499L, NSP12 P323L	20A	B.1
hCoV-19/Ethiopia/ICGEB-S6/2021	EPI_ISL_1898554	OL840807	SRX13137985	42	29,878	22,185	3.6	Spike A771S, Spike D614G, Spike S939F, N P199L, NS3 Q57H, NS3 S171L, NSP2 C487S, NSP2 F499L, NSP3 D163G, NSP3 R352H, NSP12 P323L	20A	B.1
hCoV-19/Ethiopia/ICGEB-S7/2021	EPI_ISL_1898814	OL840808	SRX13137986	43.7	29,878	22,654	4.0	Spike A520S, Spike D614G, N P199L, NS3 A110S, NS3 Q57H, NS3 S171L, NSP2 F499L, NSP5 K90R, NSP12 P323L	20A	B.1.404
hCoV-19/Ethiopia/ICGEB-S8/2021	EPI_ISL_1899047	OL840809	SRX13137987	43.7	29,874	18,443	3.0	Spike A570D, Spike D614G, Spike D1118H, Spike N501Y, Spike P330L, Spike P681H, Spike S982A, Spike T716I, Spike Y144del, N D3L, N G204R, N R203K, N S235F, NS3 T89I, NS8 K68stop, NS8 Q27stop, NS8 R52I, NS8 Y73C, NSP2 A510V, NSP3 A890D, NSP3 I1412T, NSP3 T183I, NSP12 P227L, NSP12 P323L, NSP14 P451S	20I/501Y.V1	B.1.1.7
hCoV-19/Ethiopia/ICGEB-S9/2021	EPI_ISL_1899281	OL840810	SRX13137988	43	29,877	22,240	3.8	Spike A570D, Spike D614G, Spike D1118H, Spike N501Y, Spike P681H, Spike S982A, Spike T716I, N D3L, N G204R, N R203K, N S235F, NS3 T89I, NS8 K68stop, NS8 Q27stop, NS8 R52I, NS8 Y73C, NSP3 A890D, NSP3 I1412T, NSP3 T183I, NSP12 P227L, NSP12 P323L, NSP14 P451S	20I/501Y.V1	B.1.1.7
hCoV-19/Ethiopia/ICGEB-S10/2021	EPI_ISL_1899485	OL840811	SRX13137989	45.7	29,878	19,306	3.4	Spike D614G, Spike G142A, Spike M1229I, Spike T1006I, N S194L, NS3 Q57H, NS8 E59stop, NSP2 L24F, NSP2 M117V, NSP5 S284G, NSP12 E919D, NSP12 P323L, NSP13 L297F	20A	B.1
hCoV-19/Ethiopia/ICGEB-S11/2021	EPI_ISL_2241493	OL840812	SRX13298198	43.1	29,827	5,488	1.2	Spike D614G, Spike E180Q, Spike M1229I, Spike N501Y, Spike Q183H, Spike R646P, M V23L, N D377Y, N S194L, NS3 Q57H, NS8 W45C, NSP2 L24F, NSP3 S269A, NSP3 V521I, NSP5 S284G, NSP12 L648F, NSP12 P323L, NSP13 L43F, NSP13 Y277C	20A	B.1.480
hCoV-19/Ethiopia/ICGEB-S12/2021	EPI_ISL_2241494	OL840813	SRX13298199	42.1	29,827	3,936	0.95	Spike D614G, Spike R214L, N E367G, N P199L, NS3 Q57H, NS3 S171L, NS7a R25T, NS8 T11I, NSP2 F499L, NSP4 P166S, NSP12 P323L	20A	B.1
hCoV-19/Ethiopia/ICGEB-S13/2021	EPI_ISL_2241611	OL840814	SRX13298200	43.4	29,817	3,407	0.90	Spike D614G, Spike R646P, N S202N, NS3 L108F, NS3 T223I, NS8 L84S, NS8 W45S, NSP3 P153L, NSP6 M86I, NSP12 H810Y, NSP12 K103R, NSP14 L157F	19B	A
hCoV-19/Ethiopia/ICGEB-S14/2021	EPI_ISL_2241622	OL840815	SRX13298201	44.1	29,827	2,831	0.69	Spike D614G, Spike M1229I, Spike N439K, N G179S, N S194L, NS3 Q57H, NS3 S40L, NSP2 L24F, NSP3 A1872V, NSP5 S284G, NSP8 A74V, NSP12 P323L	20A	B.1.480
hCoV-19/Ethiopia/ICGEB-S15/2021	EPI_ISL_2241623	OL840816	SRX13298202	43.4	29,827	3,129	0.80	Spike D614G, Spike T676I, N P199L, NS3 Q57H, NS3 S171L, NSP2 F499L, NSP3 V1201F, NSP5 T198A, NSP12 P323L	20A	B.1

a PANGO, Phylogenetic Assignment of Named Global Outbreak Lineages.

**FIG 1:**
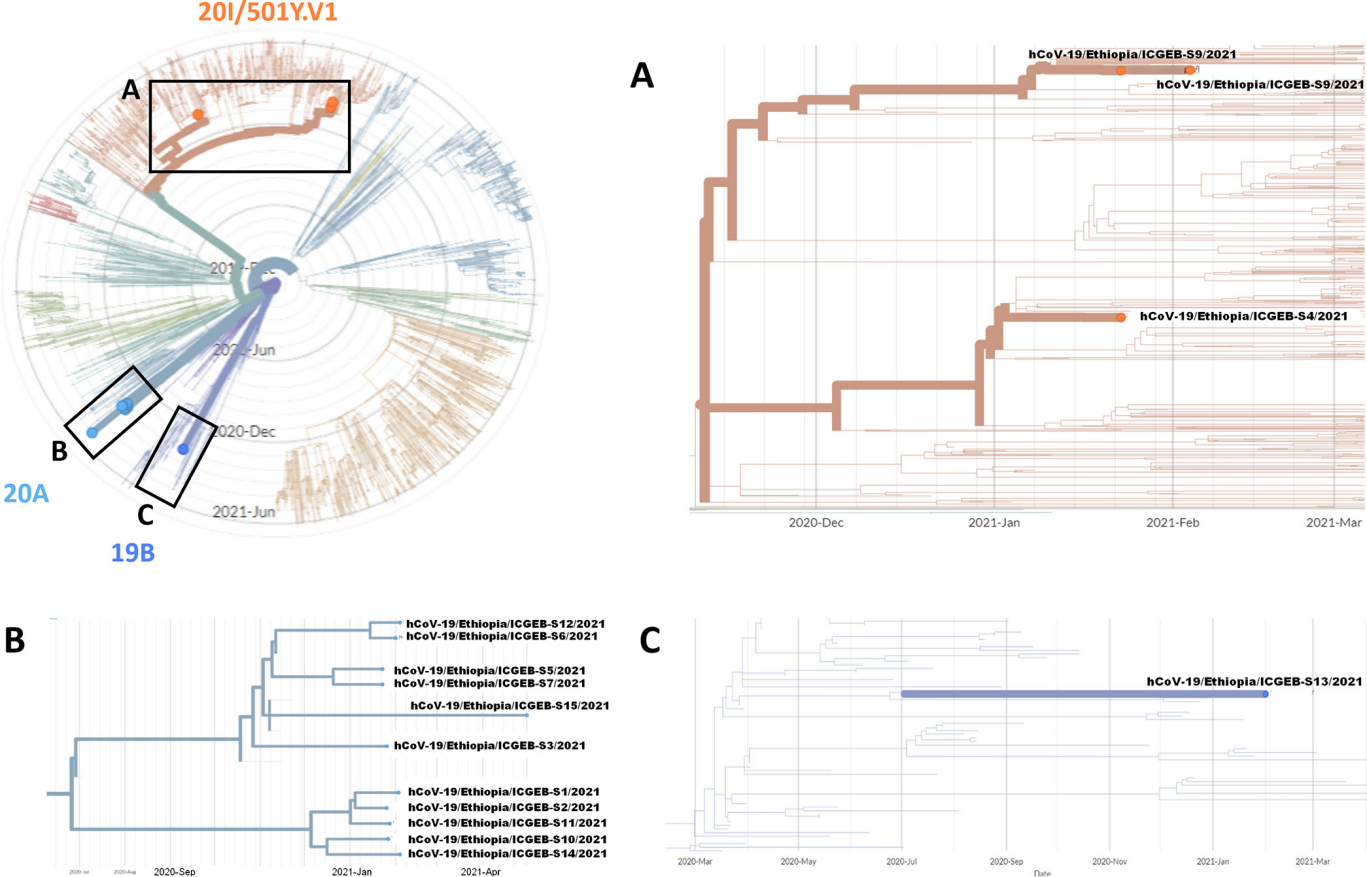
Phylogenetic analysis of sequenced SARS-CoV-2 samples from Ethiopia. Each box highlights phylogenetic relationships of samples within each lineage.

A phylogenetic analysis was conducted on the 15 sequenced samples using the Nextstrain bioinformatics platform (9, 10). The coding complete genome sequences were analyzed in the context of the Nextregions/Africa data set, which is available on GISAID (updated to 7 December 2021). An Ethiopia-focused country-level subsampling strategy was performed, using the reference strain “hCoV-19/Wuhan/WH01/2019” (EPI_ISL_402125) as the original root. Results are shown in Fig. 1. A Nextstrain analysis reveals that SARS-CoV-2 samples from Ethiopia belong to three different Nextstrain clades, as follows: 19B, 20A, and 20I/501Y.V1. A complete description of amino acidic substitutions and linages for each sample is provided in Table 1.

### Data availability.

The coding complete genome sequences and metadata of all the 15 samples were submitted to the GISAID database (www.gisaid.org) where they can be accessed through the accession numbers listed in Table 1. The coding complete genome sequences can be also accessed on the NCBI database (identifiers [IDs] OL840802 to OL840816, see Table 1); SRA accessions for each sample are available on the NCBI database under BioProject accession PRJNA780398.
